# Determination of normal values for navicular drop during walking: a new model correcting for foot length and gender

**DOI:** 10.1186/1757-1146-2-12

**Published:** 2009-05-07

**Authors:** Rasmus G Nielsen, Michael S Rathleff, Ole H Simonsen, Henning Langberg

**Affiliations:** 1 Orthopaedic Division, North Denmark Region, Aalborg Hospital, part of Aarhus University Hospital, Denmark; 2 Institute of Sports Medicine, Department of Rheumatology, Bispebjerg Hospital, Copenhagen University Hospital, Copenhagen, Denmark

## Abstract

**Background:**

The navicular drop test is a measure to evaluate the function of the medial longitudinal arch, which is important for examination of patients with overuse injuries. Conflicting results have been found with regard to differences in navicular drop between healthy and injured participants. Normal values have not yet been established as foot length, age, gender, and Body Mass Index (BMI) may influence the navicular drop. The purpose of the study was to investigate the influence of foot length, age, gender, and BMI on the navicular drop during walking.

**Methods:**

Navicular drop was measured with a novel technique (Video Sequence Analysis, VSA) using 2D video. Flat reflective markers were placed on the medial side of the calcaneus, the navicular tuberosity, and the head of the first metatarsal bone. The navicular drop was calculated as the perpendicular distance between the marker on the navicular tuberosity and the line between the markers on calcaneus and first metatarsal head. The distance between the floor and the line in standing position between the markers on calcaneus and first metatarsal were added afterwards.

**Results:**

280 randomly selected participants without any foot problems were analysed during treadmill walking (144 men, 136 women). Foot length had a significant influence on the navicular drop in both men (p < 0.001) and women (p = 0.015), whereas no significant effect was found of age (p = 0.27) or BMI (p = 0.88). Per 10 mm increase in foot length, the navicular drop increased by 0.40 mm for males and 0.31 mm for females. Linear models were created to calculate the navicular drop relative to foot length.

**Conclusion:**

The study demonstrated that the dynamic navicular drop is influenced by foot length and gender. Lack of adjustment for these factors may explain, at least to some extent, the disagreement between previous studies on navicular drop. Future studies should account for differences in these parameters.

## Background

The medial longitudinal arch (MLA) plays an important role in shock absorbance and energy transfer during walking [1,2]. Arch function depends on the shape of the foot [3], bony structure [4], ligamentous stability [5,6], and muscular fatigue [7] while factors like race [8,9], footwear [10,11], age, and gender [12] are found to influence the formation of MLA.

High-arched and low-arched foot types seem to be a risk factor for overuse injuries in sport activities. Dahle [13] found knee pain more common in football players with pronated or supinated foot types, compared with neutral foot type. Williams [14] found high-arched runners to have more ankle, bony, and lateral sided injuries, while low-arched runners had more knee, medial sided, and soft tissue injuries. Although there are trends in the literature implicating foot position, statically or dynamically, as a risk factor for exercise related injuries, Wen [15] found the literature inconclusive in a recent review. In his opinion one drawback of several studies is a failure to control for confounding variables.

Brody [16] introduced the static navicular drop test as a measure to evaluate MLA. In previous studies, mean values among healthy adults range from 3.6 to 8.1 mm in the original version of the test [17-21] and from 7.3 to 9.0 mm in modified versions [22,23]. Brody [16], Beckett [24], and Mueller [19] suggested 15, 13, and 10 mm, respectively, as the upper limit for a normal navicular drop [25]. It has shown moderate to good reliability [18,19,26], also when compared with x-ray examination [27].

Static navicular drop has been a relatively poor predictor of the navicular drop (ND) during walking [28]. A dynamic navicular drop test was introduced by Cornwall and McPoil [29] using a 6D electromagnetic motion analysis system. Among 106 healthy participants ND was found to be 5.9 mm (SD ± 2.8).

The relation between foot function and various pathologies has been examined in a few studies. Among 50 participants with ACL rupture, Beckett [24] found increased static ND (13 ± 4.4 mm, mean ± SD) compared with non-injured (6.9 ± 3.2 mm), and Reinking [30] observed a significantly increased incidence of exercise related leg pain among female athletes with a static navicular drop greater than 10 mm.

In his review Menz [25] suggested that limits of abnormal drop should be interpreted with caution, as he speculated that the ND could be influenced by foot length. This was supported by Weiner-Ogilvie and Rome [31], who proposed that an "acceptable range of normative values for clinical measurements of foot position" is needed. To our knowledge, no studies have investigated the influence of foot length, gender, age, and body mass index (BMI) on the ND during walking, which thus became the purpose of this study.

## Methods

### Participants

The study was approved by the local ethics committee (N-20070049). Informed written consent was obtained from the participants prior to the experiments. From the Danish Central Personal Register adult citizens from Aalborg Municipality were randomly selected. 320 agreed to participate. 40 participants were excluded because of overweight, age, injuries, data loss and a disability to walk on treadmill. Finally 280 healthy volunteers aged 18–68 years were included. Inclusion criteria were no lower extremity deformities or major trauma, and no pain in the lower extremity during the last three months.

### Procedures

**Figure 1:**
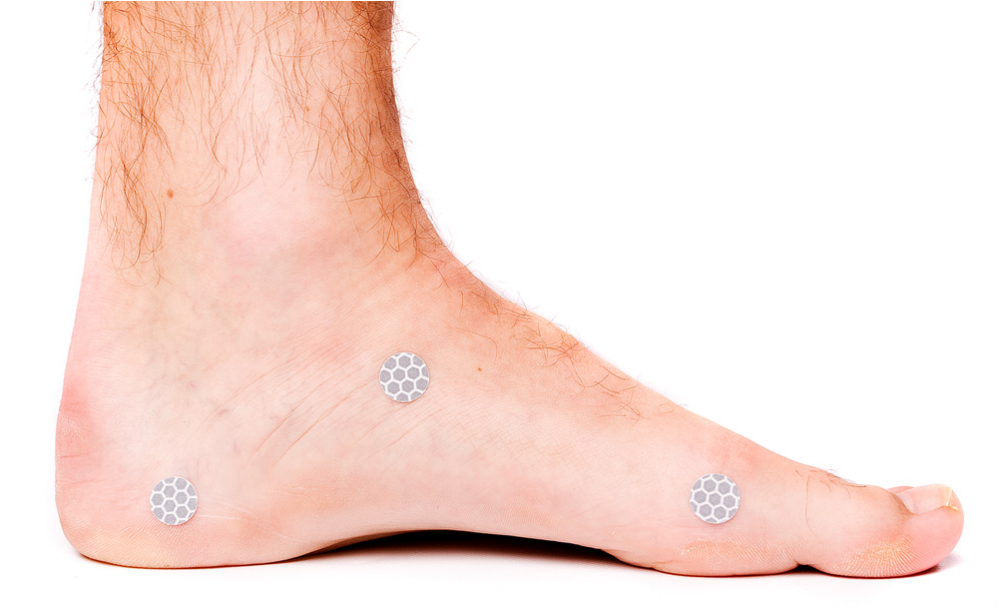
**Reflective marker positions used to calculate navicular drop (ND)**.

The foot length was measured with a ruler from the most posterior aspect of calcaneus to the tip of the longest toe. The foot length ranged from 21 – 31 cm (table 1). Custom made flat markers with a diameter of 13.5 mm made from reflective 3 M scotch tape were used. The markers were placed while participants were seated with the subtalar joint in a neutral position. Neutral position of the subtalar joint was defined as the position where talus could be palpated equally on the medial and lateral side of the foot [18]. An experienced clinician placed the markers with adhesive tape on (i) the navicular tuberosity, (ii) medial aspect of calcaneus 2 cm above the floor and 4 cm from the most posterior aspect of the calcaneus, and (iii) medial aspect of first metatarsal head 2 cm above the floor (figure 1).

**Table 1:** Demographic characteristics of the participants tested.

Parameters	All (n = 280)		Women (n = 136)	Men (n = 144)
	Mean (SD)	Range	Mean (SD)	Mean (SD)

Age (years)*	43 (31–54)	18–68	43 (34–60)	42,5 (30–54)

Height (m)	1.74 (± 0.08)	1.55–1.93	1.68 (± 0.06)	1.79 (± 0.06)

Weight (kg)	73.4 (± 12.1)	43–107	66.5 (± 9.3)	80.0 (± 10.7)

BMI (kg/m^2^)	24.2 (± 3.1)	17.6–30.5	23.5 (± 3.1)	24.8 (2.9)

Foot length (cm)	25.3 (± 1.8)	21–31	24.1 (± 1.3)	26.5 (± 1.3)

* age presented as median and interquartil range.

The participants were instructed to walk bare-footed on a treadmill at self-selected speed. After an accommodation period of six minutes [32], recordings were carried out for 20 sec.

A 2D motion capture system (VSA) was used to measure ND during walking [33]. It consists of a digital video camera (Basler Scout; Basler AG, Ahrensburg, Germany) with a 12 mm lens sampling at 86 Hz. The camera was mounted perpendicular to the sagittal plane at the level of the foot on the treadmill. ND was defined as the maximal vertical movement of the navicular bone from heel strike to the minimal height between the navicular tuberosity and the floor. It was calculated as the perpendicular distance between the marker on the navicular tuberosity and the line between the markers on calcaneus and first metatarsal head. The distance between the floor and the line in standing position between the markers on calcaneus and first metatarsal were added afterwards. ND was calculated as the mean of 20 consecutive steps.

The system was found highly reliable in a test/retest pilot study with ICC values for ND at 0.95 (within day) and 0.94 (between days).

### Statistical analysis

All data except age were parametric and therefore the Pearson product moment correlation was used. The Spearman's rank correlation was used in the analysis of age. Correlations and stepwise multiple regression techniques were applied to test for relationships between parameters. SPSS (SPSS Inc, Chicago, Illinois, USA) version 15.0 was used.

## Results

Dynamic ND ranged from 1.7 – 13.4 mm. (Table 2). 95% of the population had an ND less than 8.7 mm and greater than 1.7 mm. Approximately the same mean ND was observed among women and men (5.2 and 5.3 mm. respectively). BMI and age were found not to influence ND. (Table 3) Only foot length had an isolated significant effect on ND (p < 0.01).

**Table 2:** Mean navicular drop (ND) in men and women.

Parameters	All (n = 280)		Women (n = 136)	Men (n = 144)
	Mean ± SD	**Range**	**Mean ± SD**	**Mean ± SD**

Navicular drop (mm)	5.3 (± 1.7)	1.3 – 13.4	5.2 (± 1.6)	5.3 (± 1.8)

**Table 3:** Correlations (Pearson's r and p-value) between navicular drop (ND) and body mass index (BMI), age and foot length.

Correlations	Women	Men
BMI	-0.03 (0.77)	0.002 (0.98)

Foot length**/**cm	0.21 (0.02)	0.265 (0.001)

Age**/**years	-0.052 (0.55)	-0.12 (0.16)

By linear regression models it is shown that gender induced a modification on the effect of foot length on dynamic ND. (Table 4). The table should be read by looking at how the foot length affects ND. The B-value represents the slope of the regression, so in this case it means that the regression predicted an increase in ND in male participants of 0.40 mm (95% CI 0.19 – 0.62 mm.) every time the foot length was increased with one cm. For women the increase in ND per cm increase in foot length was 0.31 mm (95% CI 0.10 – 0.53 mm.).

**Table 4:** The influence of foot length on navicular drop (ND).

	Women (n = 136) Navicular drop (mm)			Men (n = 144) Navicular drop (mm)		
	B	95% CI	*P*	B	95% CI	*p*

Intercept	-2.34	-7.55 – 2.87	0.376	-5.36	-11.11 – 0.39	0.068

Foot length	0.31	0.10 – 0.53	0.006	0.40	0.19 – 0.62	< 0.001

**Figure 2:**
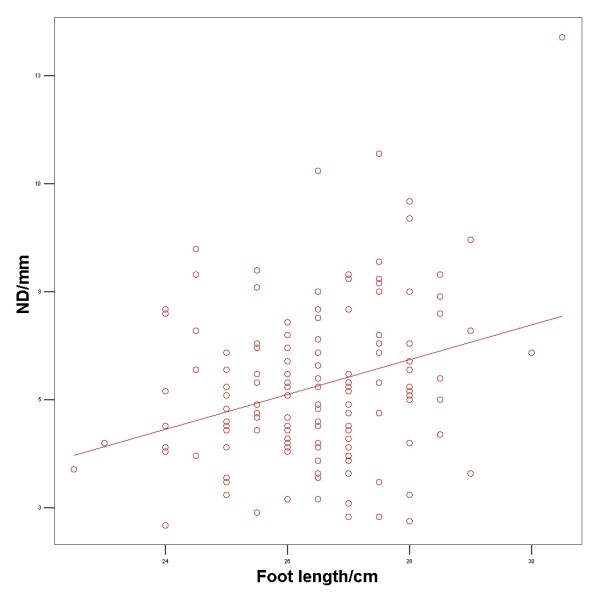
**Scatterplot showing the variation of navicular drop among male participants**.

**Figure 3:**
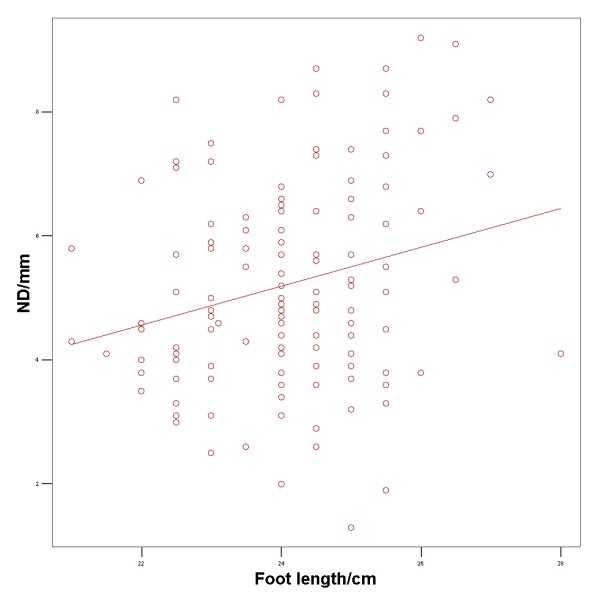
**Scatterplot showing the variation of navicular drop among female participants**.

The Pearson product moment correlation was 0.295 (p < 0.001) between foot length and ND among males and 0.241 (p = 0.005) among females. A scatter plot was created showing the variation of ND among male (figure 2) and female participants (figure 3).

## Discussion

We investigated the influence of foot length, age, gender, and BMI on the dynamic ND during walking. A positive correlation between foot length and dynamic ND was observed among these healthy participants without foot problems. 97.5% of this population had a dynamic ND of less than 8.5 mm. Thus this value could be considered as the cut-off value between normal participants and participants with abnormal ND which would correspond nicely to the 10 mm borderline suggested by Mueller [19] for the static ND. However, as the dynamic ND is influenced by foot length and the gender, the normal value for individual dynamic ND must be given relative to foot length and the gender (Figure 2 and 3). As foot length increases from 22–28 cm, the upper value (95% confident limit) of abnormal ND increases from 7.25 mm to 9.50 mm for males and from 7.8 mm to 10 mm for females.

Male participants had a mean drop of 3.9 mm with a foot length of 23 cm, while the mean drop was 6.9 mm with foot length of 30.5 cm giving a 3 mm difference between a small and a large male foot. Bandholm et al. [34] reported a significant difference in static ND of 2.8 mm between injured and healthy participants. However the static ND was not adjusted for foot length which, hypothetically, could explain the difference.

The mean ND (5.3 mm) found in present study also agrees with the mean dynamic ND (5.9 mm) found by Cornwall and McPoil [29] using an electromagnetic motion analysis system. In the present VSA system, dynamic ND was calculated as the navicular drop from heel strike to minimal navicular height, while the electromagnetic method used the difference from foot flat to heel off to calculate the "maximum vertical depression". This methodological difference may explain the 0.6 mm difference between the systems.

Measurement of the static ND might be the most appropriate technique for the clinical assessment of foot pronation [25]. Therefore simple and reliable methods to measure dynamic ND are highly warranted. Hitherto the reliability for one- and 2-D video systems has been too low for clinical and scientific purposes, and 3-D video systems are too expensive and space consuming for most clinics. The present knowledge about the relation between foot dysfunction and overuse injuries is primarily based upon 3-D video analysis. By the introduction of VSA we have demonstrated that a 2-D video system can be at least as reliable as the multiple camera systems in the traditional 3-D analysis. The 0.94 ICC for the present VSA is even higher than the 0.86 ICC found for a 3-D system with the same skin marker positioning [34]. The use of 2-D measurements in the sagittal plane corresponds well with three-dimensional measurement results in more advanced systems [35], which strengthen the method used in this study. 2-D video analysis requires one room and can be performed within a few minutes. Therefore the VSA is highly suitable for routine clinical examination and for studies requiring a large number of participants.

We found that age and BMI did not significantly influence the ND. However, we did not include participants with BMI larger than 30.5 and participants older than 68 years. Therefore it is still unknown whether BMI larger than 30.5 will influence the ND. Lai et al [36] found significant differences in ankle kinematics during walking between the obese and the non-obese participants. Likewise it remains to be studied whether age older than 68 years will influence the ND.

## Conclusion

The present study demonstrates that the dynamic navicular drop is influenced by foot length and gender. Male participants had an increase in drop of 0.40 mm every time the foot length is increased by 10 mm. The female participants had an increase of 0.31 mm every time the foot length is increased by 10 mm. Lack of adjustment for foot length and gender may cause invalid significant differences when comparing navicular drop between two groups. Future studies should adjust for foot length and gender when examining the navicular drop. For a valid comparison of participants in case-control studies we recommend matching people by foot length and gender.

## Competing interests

The authors declare that they have no competing interests.

## Authors' contributions

RGN designed the study, applied for approval by the local ethics committee (N-20070049), took part in data acquisition, and drafted the manuscript. MSR took part in data acquisition, made the statistical analysis and interpretation of data, and helped drafting the manuscript. OS and HL took part in revising the manuscript critically for important intellectual content. All authors read and approved the final manuscript.
